# Functionalized germanane/SWCNT hybrid films as flexible anodes for lithium-ion batteries†

**DOI:** 10.1039/d1na00189b

**Published:** 2021-05-17

**Authors:** Bing Wu, Jiří Šturala, Martin Veselý, Tomáš Hartman, Evgeniya Kovalska, Daniel Bouša, Jan Luxa, Jalal Azadmanjiri, Zdeněk Sofer

**Affiliations:** Department of Inorganic Chemistry, University of Chemistry and Technology Prague Technick'a 5 166 28 Prague Czech Republic zdenek.sofer@vscht.cz wui@vscht.cz; Department of Organic Technology, University of Chemistry and Technology Prague Technicka 5 166 28 Prague Czech Republic

## Abstract

Germanium, with a high theoretical capacity based on alloyed lithium and germanium (1384 mA h g^−1^ Li_15_Ge_4_), has stimulated tremendous research as a promising candidate anode material for lithium-ion batteries (LIBs). However, due to the alloying reaction of Li/Ge, the problems of inferior cycle life and massive volume expansion of germanium are equally obvious. Among all Ge-based materials, the unique layered 2D germanane (GeH and GeCH_3_) with a graphene-like structure, obtained by a chemical etching process from the Zintl phase CaGe_2_, could enable storage of large quantities of lithium between their interlayers. Besides, the layered structure has the merit of buffering the volume expansion due to the tunable interlayer spacing. In this work, the beyond theoretical capacities of 1637 mA h g^−1^ for GeH and 2048 mA h g^−1^ for GeCH_3_ were achieved in the initial lithiation reaction. Unfortunately, the dreadful capacity fading and electrode fracture happened during the subsequent electrochemical process. A solution, *i.e.* introducing single-wall carbon nanotubes (SWCNTs) into the structure of the electrodes, was found and further confirmed to improve their electrochemical performance. More noteworthy is the GeH/SWCNT flexible electrode, which exhibits a capacity of 1032.0 mA h g^−1^ at a high current density of 2000 mA g^−1^ and a remaining capacity of 653.6 mA h g^−1^ after 100 cycles at 500 mA g^−1^. After 100 cycles, the hybrid germanane/SWCNT electrodes maintained good integrity without visible fractures. These results indicate that introducing SWCNTs into germanane effectively improves the electrochemical performance and maintains the integrity of the electrodes for LIBs.

## Abstract 2

SWCNTs were introduced to develop hybrid SWCNT/germanane electrodes for lithium storage, endowing the hybrid electrodes with structural stability and enhanced electrochemical performance.

## Graphical abstract

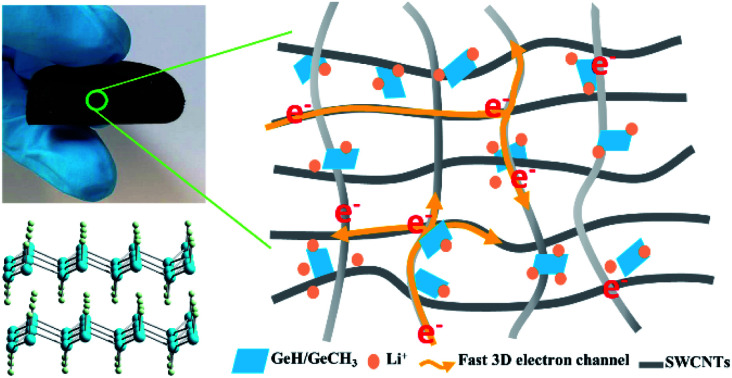

## Introduction

Lithium-ion batteries (LIBs) have become the predominant electrical energy storage devices in consumer electronic products and electric vehicles (EV) in the past decade due to the merits of stable compact energy output and long cycle life.^1^ Meanwhile, numerous materials, both as cathodes and anodes for LIBs, have been developed to endow these devices with high energy and power density as well as improved stability and lifespan. Li–Ni–Co–Al/Mn oxides have paved the way as cathode materials for high-energy-density LIBs.^2,3^ Some carbon-based materials, such as the most representative material graphite, are absolute leaders among the known anode materials for LIBs.^4–7^ Nevertheless, on account of the fact that graphite has a modest lithiation/delithiation capacity of 374 mA h g^−1^, numerous studies have been carried out to develop candidates with higher capacity than graphite. Besides, the massive consumption of graphite in commercial energy storage appliances makes it currently unsatisfactory for the ever-increasing energy-storage market. An urgent call has been made for the recycling and recovery of graphite.^8^ Seeking superior alternative anode materials to graphite is urgent and essential to meet the future demands of the colossal energy-storage market.^9,10^

Recently, many efforts have been devoted to studying lithium alloy materials from group 14 elements beyond carbon (such as Si, Ge, and Sn) due to their high specific capacities after alloying with multiple lithium ions.^11–14^ The lithiation capacity in LIBs for these candidates is unequivocal for Si (4200 mA h g^−1^ for Li_22_Si_5_), Ge (1600 mA h g^−1^ for Li_22_Ge_5_) and Sn (992 mA h g^−1^ for Li_22_Sn_5_).^1^ Among them, Si has the highest theoretical capacity. However, it has a low lithium-ion diffusivity of 10^−14^ to 10^−12^ cm^2^ s^−1^ and poor electrical conductivity of approximately 4 × 10^−4^ S m^−1^, which would cause sluggish electrochemical kinetics if it were used as an anode material for LIBs.^15^ Unlike Si, originally metallic Sn demonstrates a good electrical conductivity of approximately 9.2 × 10^6^ S m^−1^. However, the moderate theoretical capacity and low lithium-ion diffusivity (10^−16^ to 10^−14^ cm^2^ s^−1^) of Sn prevent it from being a superior candidate. In contrast, Ge has a decent specific capacity, a high electrical conductivity of 2.1 S m^−1^ (approximately 10^4^ times higher than that of Si), and fast lithium-ion diffusivity of 10^−12^ to 10^−8^ cm^2^ s^−1^, which are promising properties for LIBs with good cycling stability and excellent rate performance.^16,17^ These advantages make Ge an attractive candidate for next-generation anode materials. Nevertheless, the main drawback of Ge is the huge volume expansion (approximately 300%) during the Li-alloying process, which leads to the fracture of Ge particles and probably the delamination between the active material and current collector; these demerits would result in fast capacity degradation during long-term cycling.^14,18,19^ To address the issues arising from the volume change of Ge-based materials during alloying and dealloying reactions, many efforts were made to achieve (1) nanocrystallization in various dimensions, such as nanoparticles,^20^ nanowires,^21^ nanotubes^22^ and nano-Ge film;^23^ (2) a porous structure;^24–26^ and (3) coating or combining with carbon materials, such as amorphous carbon,^27^ graphene^28^ or carbon nanotubes,^29^ as sufficient matrix buffers.

Graphite has different lithiation and delithiation mechanisms compared to lithium-containing materials of group 14 (Si, Ge and Sn) in LIBs due to the layered honeycomb structure and weak interlayer bonds. In addition, it has the ability to reduce the volume expansion upon the intercalation of lithium into the interlayer, which brings long-term cycling stability. Drawing inspiration from this, many two-dimensional (2D) van der Waals materials were developed as lithium alloying materials for LIBs in recent years.^30,31^ Recently, Ge-based layered materials with a buckled graphene-like structure were obtained by a topotactic deintercalation of calcium from the corresponding Zintl phase CaGe_2_.^32–35^ The materials have been found to be favourable for numerous applications ranging from electronic^36^ to optical,^37^ transistor^38^ and energy storage^39,40^ devices due to the unique chemical and physical properties caused by tunable thickness from few-layer to ‘bulk’ and various functional groups attached to the Ge surface (such as in germanane (Ge_6_H_6_) or methyl germanane (Ge_6_(CH_3_)_6_)). These materials based on elements from group 14 represent quite novel substrates for LIBs. And their electrochemical behaviour as anodes is far less studied and it is still a challenge for them to meet the specific demands of the energy storage sector.

In this work, flexible binder-free anodes composed of single-wall carbon nanotubes (SWCNTs) and germanane (GeH/SWCNTs) or methyl germanane (GeCH_3_/SWCNTs) are facilely fabricated. SWCNTs serve both as a flexible buffer matrix, which prevents the volume expansion of Ge-based materials, and a 3D fast conductive network of electrons. The obtained results during battery testing demonstrate the improved electrochemical performance of GeH/SWCNT and GeCH_3_/SWCNT electrodes compared to the pure GeH and GeCH_3_ electrodes. In addition, the SWCNT-incorporated flexible electrodes demonstrate excellent mechanical strength in maintaining the integrity of the electrodes during long-term cycling. In contrast, the pure GeH and GeCH_3_ electrodes were cracked and delaminated from the current collector due to the volume expansion of germanium upon cycling. Our results show that hybrid composites made of 2D Ge-based materials and SWCNTs effectively improve the electrochemical performance and electrode stability of LIBs.

## Experimental section

### Chemicals

Single wall carbon nanotubes (SWCNTs, OCSiAl, 80%); *N*-methylpyrrolidone (NMP, Sigma-Aldrich, 99.7%); poly(vinylidene fluoride) (PVDF, Alfa Aesar); ethylene carbonate (EC, supplier); dimethyl carbonate (DMC, supplier); LiPF_6_ (supplier); carbon black (Cabot Corporation); Ge_6_H_6_ and Ge_6_(CH_3_)_6_ (obtained by the topotactic reaction from CaGe_2_ accordingly).^40,41^

### Materials characterization

The morphology and element compositions were examined using a Scanning Electron Microscope (SEM, Tescan MAIA 3, Czech Republic) equipped with an Energy Dispersive Spectrometer (EDS, Oxford Instruments, England). The phase evolution and crystal structure were characterized using an X-ray diffractometer (XRD, Bruker D8 with Cu Kα radiation, Germany) and transmission electron microscopy (TEM, EFTEM Jeol 2200 FS microscope, Japan). Thermogravimetric analysis (TGA) was used to characterize the thermal properties of the material (heating rate 10 °C min^−1^, helium flow rate of 100 mL min^−1^, Setaram). The analysis of surface composition was performed by X-ray photoelectron spectroscopy (XPS, SPECS, Germany). The vibration mode of the materials was confirmed by Raman spectroscopy (using a 532 nm laser, Renishaw, England) and FTIR (FTIR-ATR, NICOLET iS50R, Czech Republic).

### Preparation of the free-standing GeH/SWCNT and GeCH_3_/SWCNT electrodes

The SWCNTs before being used were preliminarily purified and slightly oxidized by reaction with KMnO_4_ in the presence of concentrated sulfuric acid. To obtain the target electrode for LIBs, at first, 0.01 mg SWCNTs was sonicated and dispersed for 1.5 hours in NMP (30 mL). Then, GeH or GeCH_3_ (0.09 mg) was introduced into the above dispersion with mild sonication for 30 min. This was followed by vacuum-filtration through a polyamide filter (0.45 μm pore size) and drying *in vacuo*. The as-obtained GeH/SWCNT and GeCH_3_/SWCNT hybrid films were punched into a diameter of 10 mm as anodes for LIBs.

For comparison, a slurry consisting of 75% pure active material (GeH or GeCH_3_), 15% carbon black, 10% PVDF, and NMP solution was coated on copper foil. The slurry on copper foil was dried at 60 °C under an argon atmosphere overnight, and then punched into electrodes of 10 mm diameter.

### Lithium-ion battery assembly and performance characterization

The prepared electrodes were assembled into a CR2032 coin cell using lithium foil as a counter electrode and porous polypropylene membrane (Celgard 2400) as a separator; the LIB fabrication was carried out in an argon-filled glovebox. 1 M solution of LiPF_6_ in a mixture of EC and DMC (1 : 1 v/v) served as an electrolyte. The discharge/charge performance analysis was conducted on a Neware battery test system (Neware BTX 7.6, Shenzhen, China) in a fixed potential window from 0.001 to 3.0 V (*vs.* Li^+^/Li). The cyclic voltammetry (CV) measurements and Electrochemical Impedance Spectroscopy (EIS) measurements were conducted using an Autolab PGSTAT204 (Eco Chemie, Utrecht, The Netherlands) workstation.

## Results and discussion

**Fig. 1:**
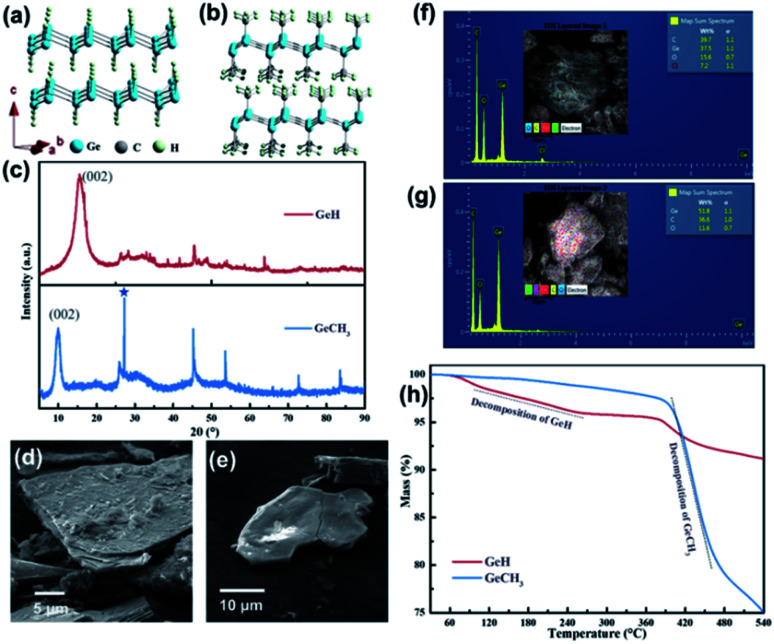
Structure and chemical composition analysis of GeH and GeCH_3_: (a and b) corresponding crystal structures; (c) XRD patterns; (d and e) SEM images; (f and g) elemental analysis; (h) TG curves.

The schematic structures of layered GeH and GeCH_3_ are presented in Fig. 1a and b, where the sheets composed of Ge_6_ rings were functionalized by –H and –CH_3_ groups, respectively. X-ray diffraction (XRD) was used to confirm the crystal structure of the fabricated GeH and GeCH_3_ (Fig. 1c), which was in good agreement with previously published results.^42,43^ The prominent peaks at about 15.56° and 9.89° corresponding to the (002) plane of GeH and GeCH_3_ indicate their interlayer spacing of 5.7 and 9.0 Å. Compared to GeH, the 1.5 times increased interlayer distance of GeCH_3_ might be ascribed to the bigger size of the –CH_3_ group. However, GeCH_3_ contains cubic germanium after the exfoliation as labelled by the asterisk at 27.3° in Fig. 1c.^42^ It has to be noted that both groups (–H and –CH_3_) cause a larger interlayer distance between Ge sheets than graphite (3.4 Å).^44^ This will lead to an enhanced electrochemical performance by reducing the migration resistance of lithium ions in host materials during charge and discharge cycling. The electron diffraction in Fig. S1† can be indexed to the hexagonal cell of layered Ge materials. The SEM images of GeH and GeCH_3_ sheets are presented in Fig. 1d and e, showing a layer stacking structure. EDS analysis (Fig. 1f and g) was used to determine the impurity of elements, from which we observe slight oxidation of the prepared material. The traces of Cl element remaining in the GeH sample can be ascribed to the use of HCl during the preparation of GeH from CaGe_2_. The thermal properties of GeH and GeCH_3_ are shown in Fig. 1h. The initial decomposition of GeH starts at about 120 °C and is finished at about 250 °C followed by further decomposition, which begins at about 375 °C. GeCH_3_ starts to decompose at about 380 °C indicating its higher thermal stability.^39^

**Fig. 2:**
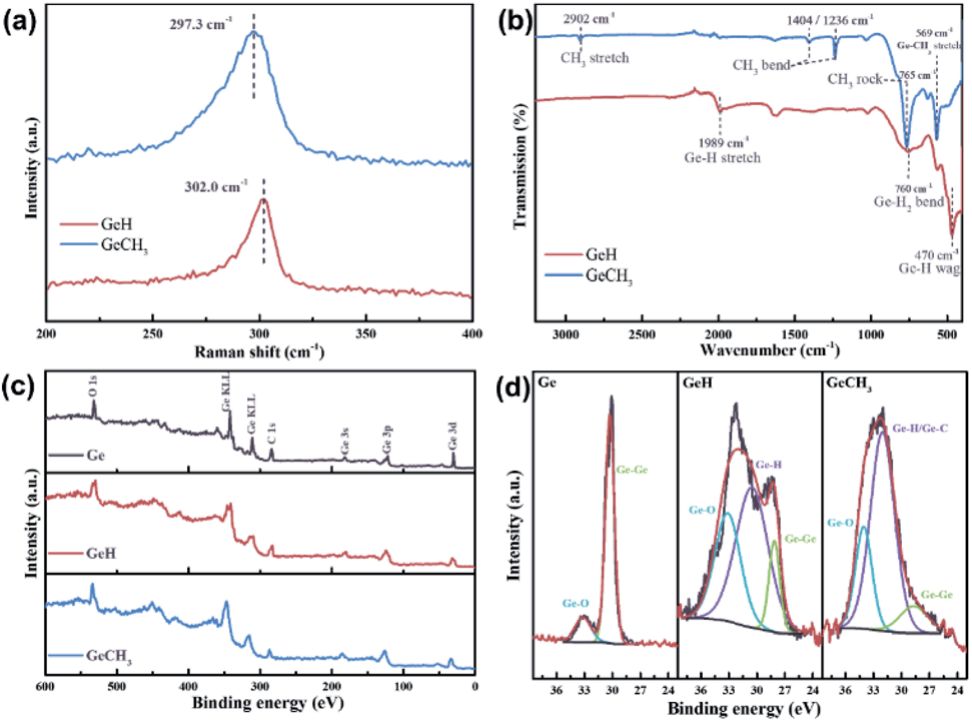
(a) Raman spectra showing the in-plane vibration and (b) FTIR spectral analysis of GeH and GeCH_3_. XPS of Ge, GeH and GeCH_3_ corrected by C 1s at 284.8 eV: (c) survey spectra and (d) high-resolution spectra of Ge 3d.

Raman, FTIR and XPS further identified the structure and chemical characteristics of the as-prepared GeH and GeCH_3_. The Raman spectra in Fig. 2a exhibit E_2_ modes of in-plane Ge–Ge lattice vibration at 302 cm^−1^ and 297 cm^−1^ for GeH and GeCH_3_, respectively, which agrees with the reported results.^45,46^ The surface terminations of –H and –CH_3_ in GeH and GeCH_3_ were further determined by FTIR as shown in Fig. 2b. In GeH, the peaks at 1989 cm^−1^, 760 cm^−1^ and 470 cm^−1^ could be identified as the Ge–H stretching, Ge–H_2_ bending and Ge–H wagging modes, respectively.^45^ In GeCH_3_, the –CH_3_ stretching mode is observed at 2902 cm^−1^, the –CH_3_ bending at 1404 and 1236 cm^−1^, the –CH_3_ rocking at 765 cm^−1^ and Ge–C stretching at 569 cm^−1^.^46^ XPS was employed to explore the surface composition and chemical state of GeH and GeCH_3_. The XPS survey spectra shown in Fig. 2c confirmed the existence of Ge, C and O. Fig. 2d shows the high-resolution Ge 3d XPS spectra of Ge (left), GeH (middle) and GeCH_3_ (right), corrected by C 1s at 284.8 eV. All the prepared samples were oxidized to some extent, which is inevitable during sample preparation. Compared to metalloid Ge, the Ge 3d XPS spectra are fitted with the Ge–H bond at 30.5 eV and Ge–H/Ge–C bonds at 32.0 eV for the as-prepared GeH and GeCH_3_, respectively. Meanwhile, spin–orbit coupling of Ge 3d was neglected.^46,47,48^ These results confirmed the presence of –H and –CH_3_ functional groups confirming the surface termination of germanium.

**Fig. 3:**
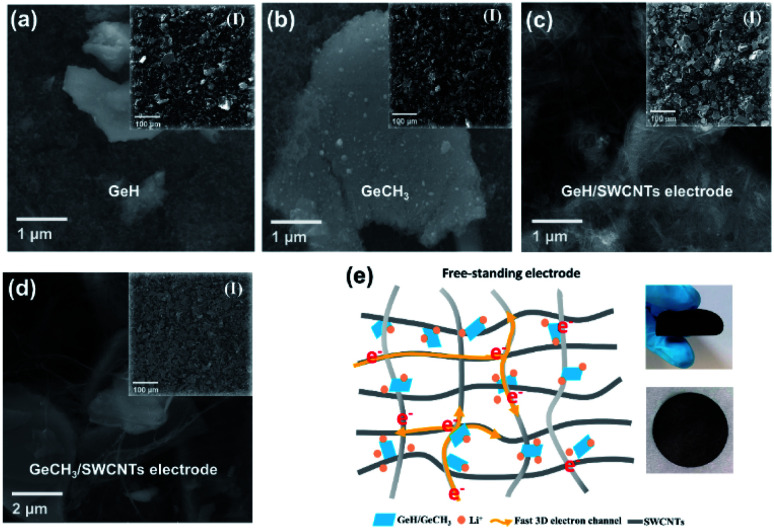
SEM images of the as-prepared electrodes for LIBs: traditional electrodes made of (a) GeH and (b) GeCH_3_; free-standing electrodes without any binder named (c) GeH/SWCNT and (d) GeCH_3_/SWCNT electrodes. (e) Schematic of electronic transportation in a SWCNT matrix (left) and the corresponding photographs of the flexible SWCNT-based electrode (right).

To evaluate the electrochemical performances, both pure and SWCNT-containing functionalized germanane were used to prepare anodes *versus* metallic Li in coin cells. The SEM images shown in Fig. 3a–d exhibit the morphology of the prepared electrodes. For pure GeH (Fig. 3a) and GeCH_3_ (Fig. 3b) electrodes, the binder and conductive carbon black were mixed with active materials. Meanwhile, in hybrid GeH/SWCNT (Fig. 3c) and GeCH_3_/SWCNT (Fig. 3d) electrodes, GeH and GeCH_3_ are covered and restrained by the crosslinking SWCNTs, which has the merits of affording a 3D fast conductive network and buffering the volume expansion of the Ge-based material. Fig. 3e shows the schematic of hybrid Ge/SWCNT flexible electrodes (left) and the corresponding photographs (right).

**Fig. 4:**
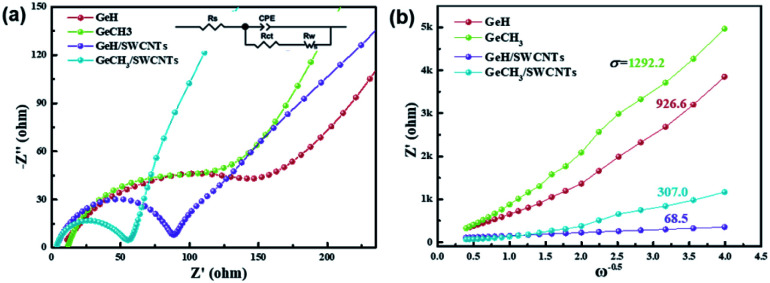
(a) Electrochemical impedance spectral (EIS) analysis of the as-prepared Ge-based electrodes; (b) the relationship plot between *Z*′ and *ω*^−0.5^ derived from the low-frequency region of the corresponding EIS spectra.

The electrode kinetics for the as-prepared samples were studied by AC impedance analysis and Nyquist plots (Fig. 4a). The semicircle diameter in the high-frequency region indicates the charge-transfer resistance (*R*_ct_) at the electrode/electrolyte interface. The pure GeH and GeCH_3_ electrodes show a bigger semicircle than the SWCNT incorporated electrodes; this indicates that the incorporation of SWCNTs has efficiently decreased the *R*_ct_ of GeH/SWCNT and GeCH_3_/SWCNT electrodes. The slope in the low-frequency region represents the Warburg impedance *Z*′, which is associated with the diffusion of Li^+^ into the active materials. The diffusion coefficient *D*_Li^+^_ can be evaluated based on the slope of *Z*′ ∼ *ω*^0.5^ (Fig. 4b) according to the following equation:^47^
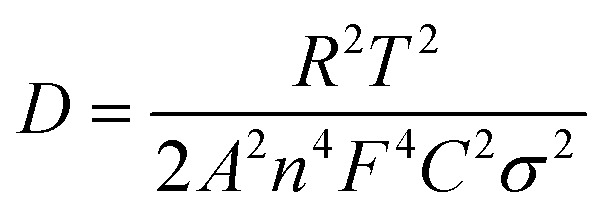
where *R* (8.314 J K^−1^ mol^−1^) is the universal gas constant, *T* is the experimental thermodynamic temperature at 298 K, *A* is the surface area of the as-prepared electrode, *n* is the number of the electrons per molecule during the electron transfer reaction (*n* = 4.4 for Li_22_Ge_5_), *F* (96 500 C mol^−1^) is the Faraday constant, *C* is the concentration of lithium ions, and *σ* is the Warburg factor which has a relationship with *Z*′ (*ω* = 2π*f*), where *Z*′ = *R* + *σω*^−0.5^. Thus, *D*_Li^+^_ is inversely proportional to the slope of *σ*^2^. Based on the values of the slope shown in Fig. 4b, the diffusion coefficient of Li^+^ was improved 183 times for GeH/SWCNT hybrid films compared to the pure GeH electrode, and 17.7 times for GeCH_3_/SWCNTs compared to the pure GeCH_3_ electrode.

**Fig. 5:**
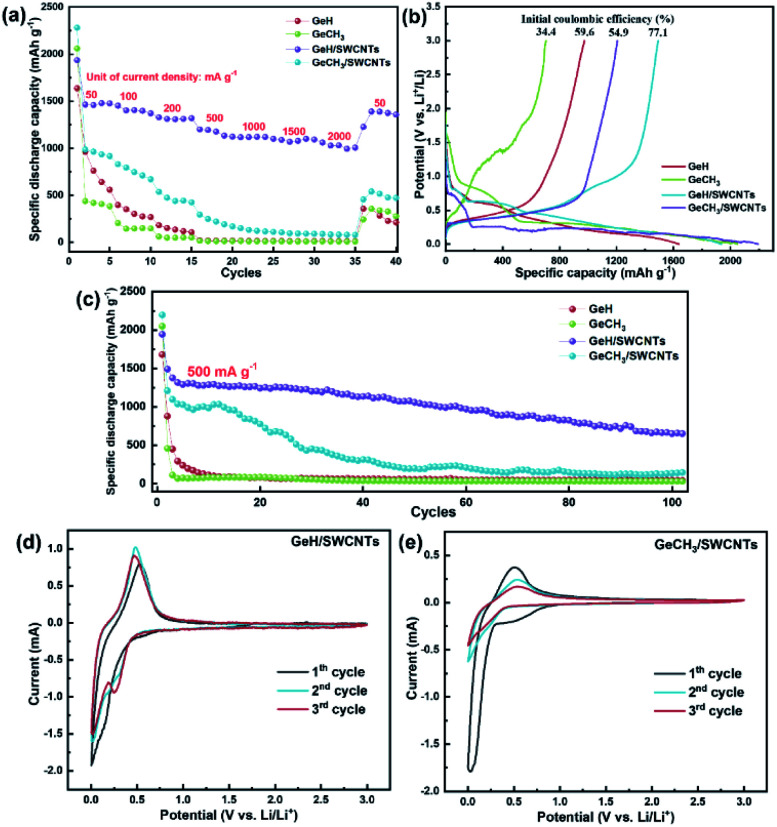
Electrochemical cycling stability analysis of the as-prepared electrodes: (a) rate performance at different current densities; (b) initial C/DC curves; (c) long-term cycling performance under 500 mA g^−1^; cyclic voltammetry curves of (d) GeH/SWCNTs and (e) GeCH_3_/SWCNTs at a scan rate of 0.2 mV s^−1^.

The rate performance of the as-prepared electrodes was tested at increasing current densities from 50 mA g^−1^ to 2000 mA g^−1^, and then restored to 50 mA g^−1^, with five cycles at each current density. The cycle performance of SWCNTs is shown in Fig. S3a and b.† After the initial activation process, SWCNTs exhibited a reversible specific capacity of ∼420 mA h g^−1^, which means that the capacity contribution of SWCNTs is ∼42 mA h g^−1^ based on the 10 wt% SWCNTs in our prepared electrode. The results shown in Fig. 5a revealed that the reversible capacity decreases for all samples as the current density is increased. The samples combined with SWCNTs demonstrate the improved reversible specific capacity in comparison to their pure counterparts. The initial discharge capacities are 1637 mA h g^−1^ for GeH, 2048 mA h g^−1^ for GeCH_3_, 1933 mA h g^−1^ for GeH/SWCNTs and 2198 mA h g^−1^ for GeCH_3_/SWCNTs. The corresponding charge and discharge (C/DC) curves at each current density are shown in Fig. S3c and d.† It is most noteworthy that the GeH/SWCNT electrode still maintains the best cycling stability at each current density among all the prepared electrodes. It can realize a specific discharge capacity of approximately 1000 mA h g^−1^ even at a large current density of 2000 mA g^−1^. Unfortunately, the specific capacity of GeCH_3_/SWCNTs falls rapidly after the 1st C/DC cycle. When the current density was restored to the initial value of 50 mA g^−1^, the GeH/SWCNT electrode exhibited a specific capacity of 1388 mA h g^−1^ with approximately 94% retention, indicating excellent electrochemical stability upon cycling at large current density.

It is known that the 1st discharge process for Li^+^ intercalation into Ge-based anode materials is accompanied by the formation of a solid electrolyte interphase (SEI) layer on the surface of the active material.^43^ The SEI layer is composed of lithium-carbonate byproducts from the decomposition of the electrolyte, which can consume lithium ions in the batteries, resulting in the decrease of the initial coulombic efficiency. On the other hand, the SEI layer can prevent the active material from further eroding during long-term electrochemical cycling, improving the cycling stability.^49^Fig. 5b shows the initial C/DC curves of the as-prepared samples. The discharge plateau around 0.7 V (*vs.* Li/Li^+^) is ascribed to the formation of an SEI layer.^50^ GeH/SWCNTs had the most prolonged discharge plateau at this voltage range, indicating the formation of the thicker SEI layer.^51^ In addition, the introduction of SWCNTs increased the initial coulombic efficiency from 59.6% to 77.1% for GeH and from 34.4% to 54.9% for GeCH_3_.

To further evaluate the electrochemical performance of the prepared Ge-based material, long-term cycling for 100 cycles at a current density of 500 mA g^−1^ was performed for each material. As shown in Fig. 5c, the specific capacity decreases rapidly to approximately 100 mA h g^−1^ after several cycles for the pure GeH and GeCH_3_ electrodes. Though the GeCH_3_/SWCNT hybrid electrode shows an improvement in capacity and cycling stability compared to pure GeCH_3_, only for 15 cycles are capacities over 1000 mA h g^−1^ maintained, and the value decreases below 300 mA h g^−1^ after 30 cycles. Moreover, GeH/SWCNTs, with excellent rate performance, still exhibit the most outstanding cycling stability among all the samples, with a discharge specific capacity of 653.6 mA h g^−1^ after 100 cycles. Moreover, Fig. S3e† shows the cycling performance of hybrid electrodes under a small current density of 200 mA g^−1^. Both GeH/SWCNTs and GeCH_3_/SWCNTs exhibit more stable cycling stability than at 500 mA g^−1^. In particular, GeH/SWCNTs retain a high capacity of 1188.2 mA h g^−1^ after 70 cycles with a capacity degradation rate of 0.24% per cycle.

The crystalline structure of the hybrid electrodes was characterized by XRD, as shown in Fig. S4a.† Compared with the XRD patterns before cycling, the electrode materials became structurally amorphous. On the other hand, the reaction region of the lithium counter electrode shown in Fig. S4b and c† was interspersed by some black material, which can be ascribed to the penetration of the germanium compounds from the anode electrode. The structural evolution and depletion of the active material may contribute to continuous capacity decay for hybrid electrodes.

CV measurements at a scan rate of 0.2 mV s^−1^ were conducted for hybrid germanane/SWCNT electrodes to demonstrate the lithium storage mechanism. As shown in Fig. 5d and e, GeH/SWCNTs and GeCH_3_/SWCNTs displayed broad irreversible reduction peaks at 0.6 V during the 1st cycle, contributing to the formation of a solid electrolyte interface (SEI). The reduction peak of GeH/SWCNTs at 0.16 V for the first cycle shifted to 0.25 V in the continuous scan, while the reduction peak of GeCH_3_/SWCNTs at 0.07 V for the first cycle moved to 0.20 V in the subsequent sweep. These reduction peaks can be ascribed to the intercalation of lithium ions into the layered germanane and the formation of lithium-intercalated GeH and GeCH_3_. The reversible oxidation peaks at ∼0.48 V for GeH/SWCNTs and at ∼0.52 V for GeCH_3_/SWCNTs belong to the delithiation or dealloying reaction. During subsequent cycling for the as-prepared germanane, the observed pair of redox peaks indicates that the single delithiation/lithiation or dealloying/alloying reaction dominates the following electrochemical process.

**Fig. 6:**
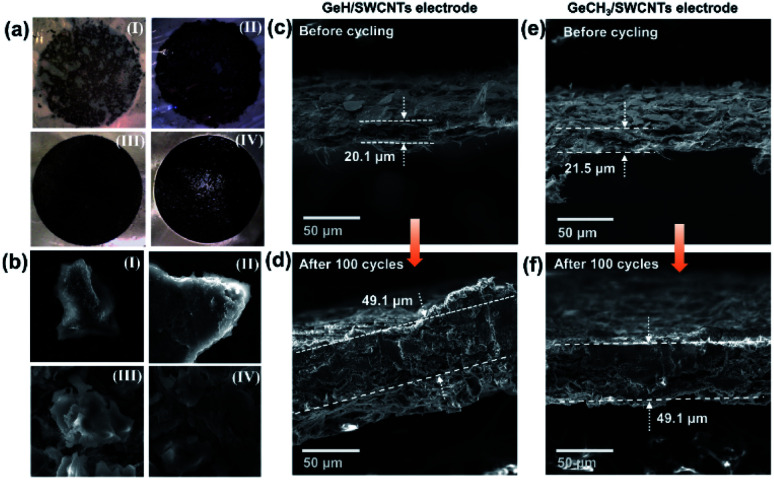
(a) Photographs of the as-prepared electrodes after 100 cycles at 500 mA h g^−1^: (I) pure GeH electrode, (II) pure GeCH_3_ electrode, (III) GeH/SWCNT hybrid electrode and (IV) GeCH_3_/SWCNT hybrid electrode; (b) the corresponding SEM images of the materials from (a); thickness before and after cycling: (c and d) GeH/SWCNT electrode; (e and f) GeCH_3_/SWCNT electrode, respectively.

To investigate any structural change of the anode material, the cells were disassembled inside an argon-filled glovebox after 100 cycles, and the electrodes were washed with DMC. Fig. 6a shows the images of the cycled electrodes. Both pure GeH (a-I) and GeCH_3_ (a-II) electrodes exhibited visible cracks and delamination from the current collector due to the volume expansion–compression during lithiation/delithiation cycles. However, no obvious defects were found in the GeH/SWCNT (III) and GeCH_3_/SWCNT (IV) hybrid electrodes; this indicates that incorporation of SWCNTs improves the mechanical strength and maintains the integrity of the electrodes. SEM was further used to investigate the corresponding electrodes as shown in Fig. 6b. The outlines of GeH and GeCH_3_ particles are misshapen and covered by an inhomogeneous SEI layer in the pure GeH and GeCH_3_ electrodes. At the same time, clean and soft surface structures are exhibited in the hybrid electrodes under the protection of SWCNTs. SEM images further evaluated the volume changes of the electrodes after 100 cycles. Due to the huge volume expansion, the pure GeH and GeCH_3_ electrodes were severely cracked and delaminated from the current collector, as shown in Fig. S5,† and their cross-sections could not be measured by SEM. As shown in Fig. 6c–f, both GeH/SWCNTs and GeCH_3_/SWCNTs exhibited approximately 2.5 times volume expansion; however, the hybrid electrodes can still maintain their integrity at such a massive rate of volume expansion without visible defects due to the flexible SWCNTs.

## Conclusions

In summary, layered germanium-based materials (GeH and GeCH_3_) obtained by a topological reaction from the Zintl phase CaGe_2_ with 36% HCl at −20 °C or with CH_3_I at room temperature were combined with SWCNTs to form flexible free-standing anodes for LIBs. Compared with pure GeH and GeCH_3_ electrodes, the corresponding SWCNT-incorporated hybrid electrodes exhibited improved kinetic performance, initial coulombic efficiency, rate performance and cycling stability. It is worth noting that the GeH/SWCNT electrode can reach a discharge capacity of 1000 mA h g^−1^ at a high current density of 2000 mA g^−1^ and achieve 653.6 mA h g^−1^ after 100 cycles at 500 mA g^−1^. The SEM analysis of the cycled electrodes showed that the incorporation of SWCNTs suppressed the negative effect of the enormous volume expansion of the Ge-based material due to the enhanced mechanical strength of the electrodes. In contrast, the pure GeH and GeCH_3_ electrodes in the absence of SWCNTs were cracked and delaminated from the current collector after long-term cycling. We believe our investigation will pave the way for further understanding of the electrochemical behavior of new layered germanium-based materials and speed up the development of high-performance anodes for LIBs.

## Author contributions

The manuscript was written by contribution from all authors.

## Conflicts of interest

There are no conflicts to declare.

## Supplementary Material

NA-003-D1NA00189B-s001
