# Supplementary material for: Functionalized germanane/SWCNT hybrid films as flexible anodes for lithium-ion batteries
Source: Nanoscale Adv. 2021 May 17;3(15):4440–6. doi: 10.1039/d1na00189b (PMC9418117; doi:10.1039/d1na00189b)
Supplement: NA-003-D1NA00189B-s001 [file NA-003-D1NA00189B-s001.pdf]

Electronic Supplementary Material (ESI) for Nanoscale Advances.
This journal is © The Royal Society of Chemistry 2021

Supporting information

Functionalized Germanane/SWCNTs Hybrid Films as Flexible Anodes for Lithium-ion Batteries

Bing Wu, Jiří Šturala, Martin Veselý, Tomáš Hartman, Evgeniya Kovalska, Daniel Bouša, Jan Luxa, Jalal Azadmanjiri, Zdenek Sofer**

Department of Inorganic Chemistry, University of Chemistry and Technology Prague, Technická 5, 166 28 Prague 6, Czech Republic

*Corresponding author. E-mail: soferz@vscht.cz ; wui@vscht.cz

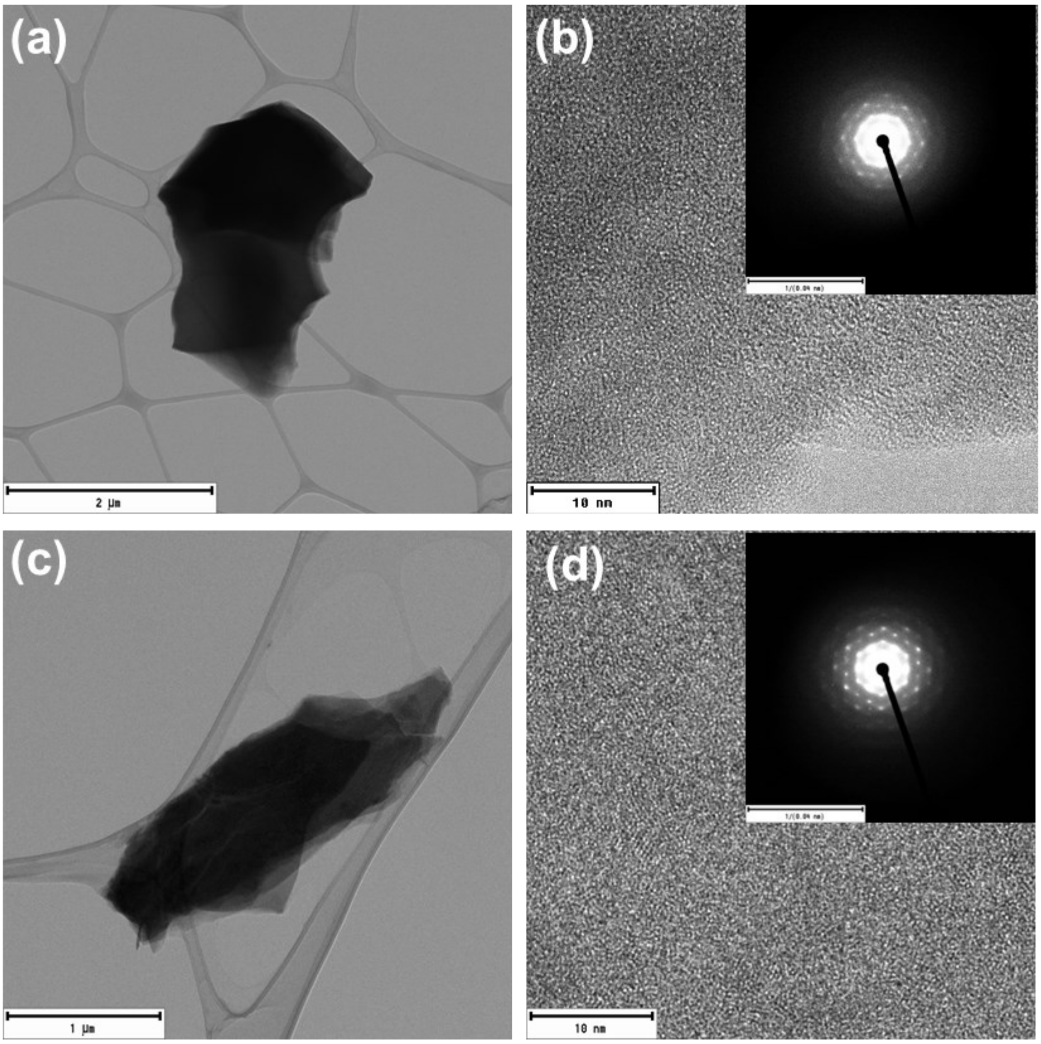

Figure S1. TEM and SAED of GeH (a-b) and $GeCH_3$ (c-d)

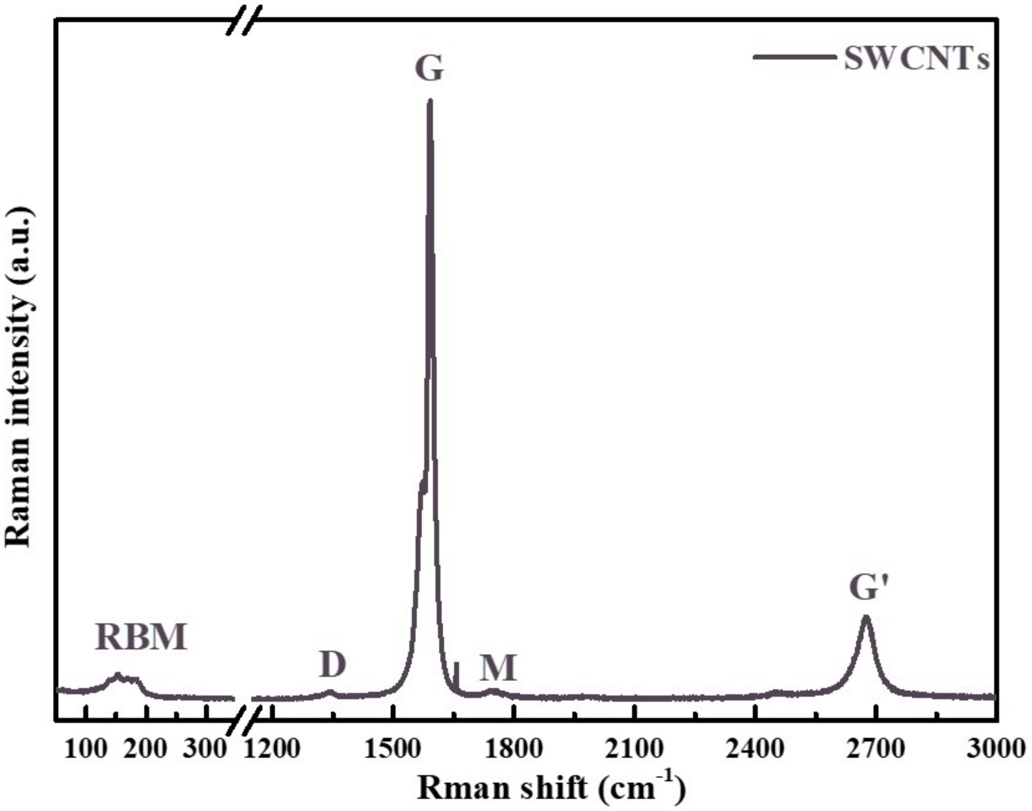

Figure S2. The Raman spectrum of SWCNTs.

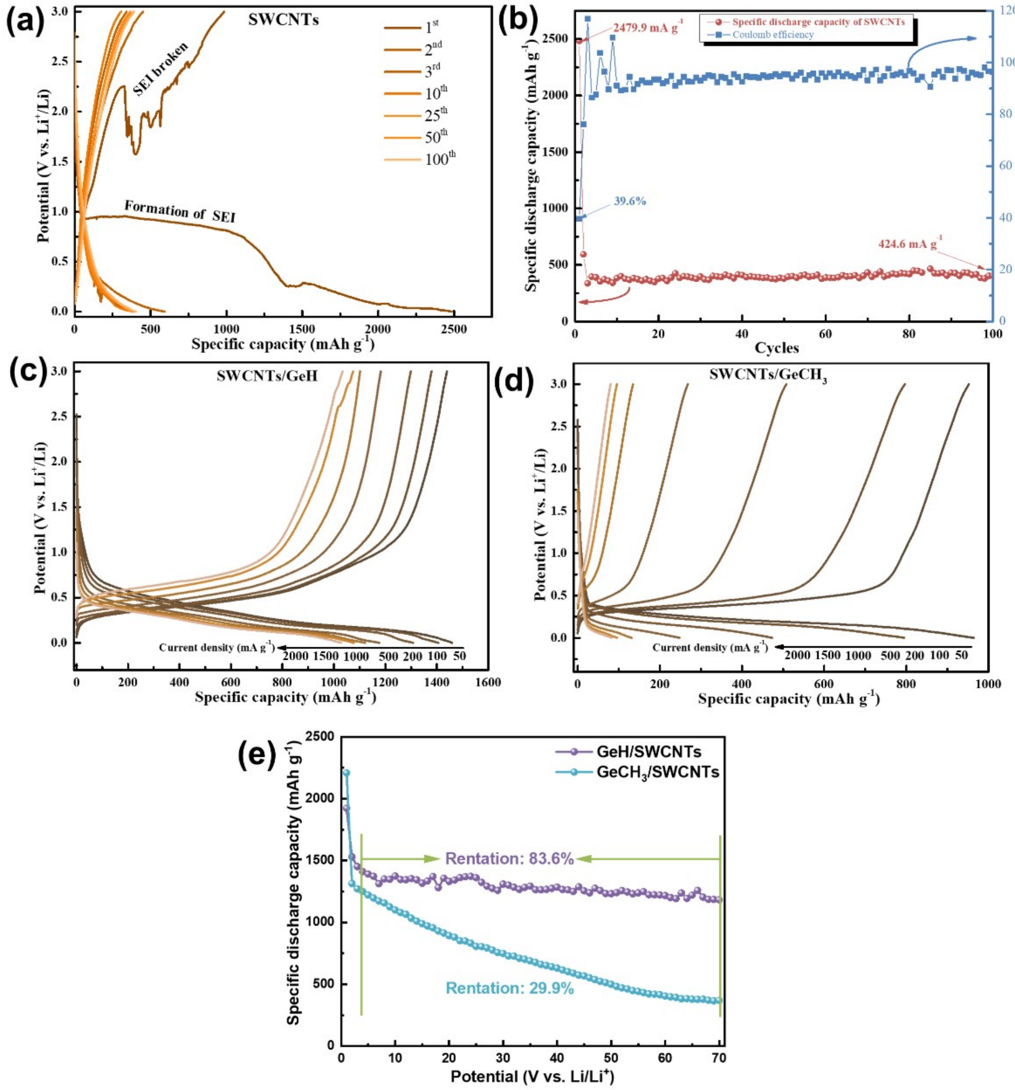

Figure S3. (a) and (b) Cycling performance of SWCNTs; C/DC curves of (e) GeH/SWCNTs and (f) $GeCH_3$/SWCNTs at various current density; (e)cycling performance of GeH/SWCNTs and $GeCH_3$/SWCNTs electrode at 200 mA g^{-1}.

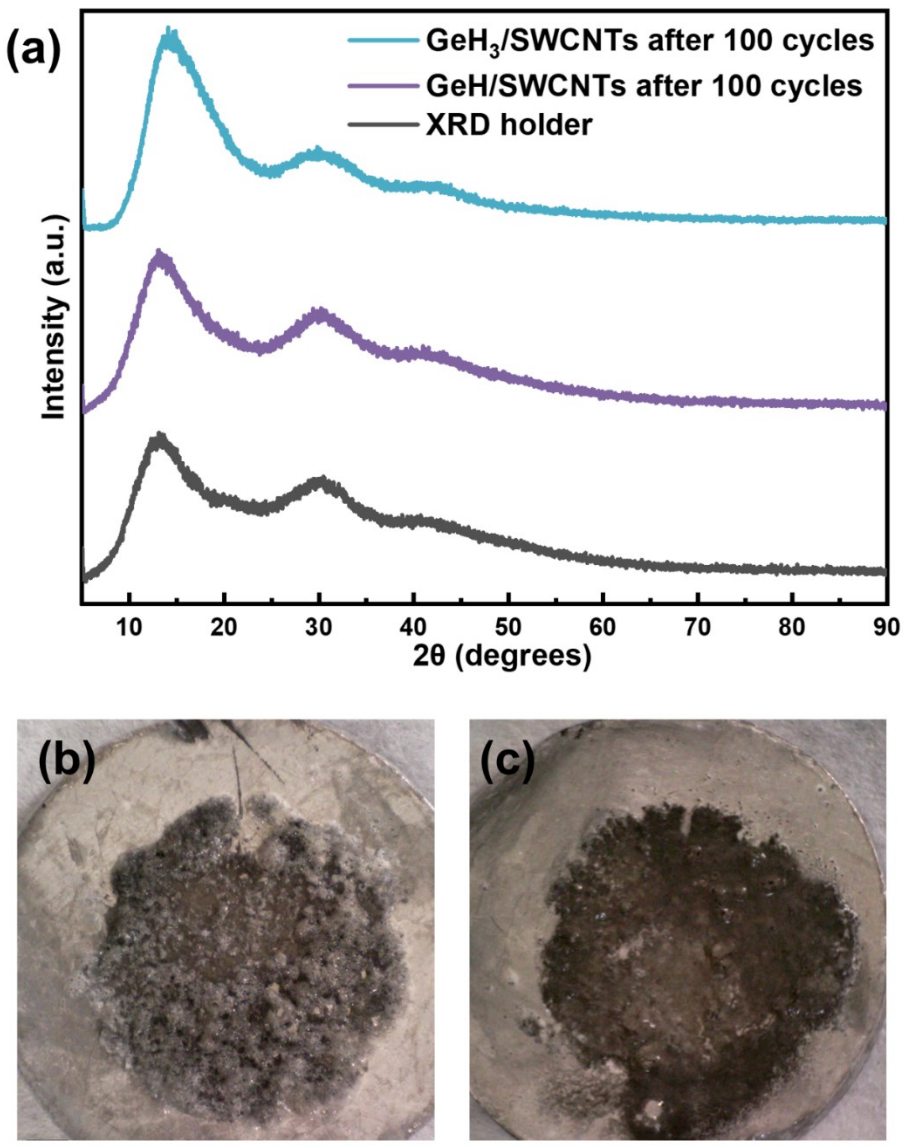

Figure S4. (a) XRD patterns of GeH/SWCNTs and $GeCH_3$/SWCNTs electrode after 100 cyclings at 500 mA g^{-1}; and the corresponding counter lithium-metal electrodes: (b) GeH/SWCNTs; (c) $GeCH_3$/SWCNTs.

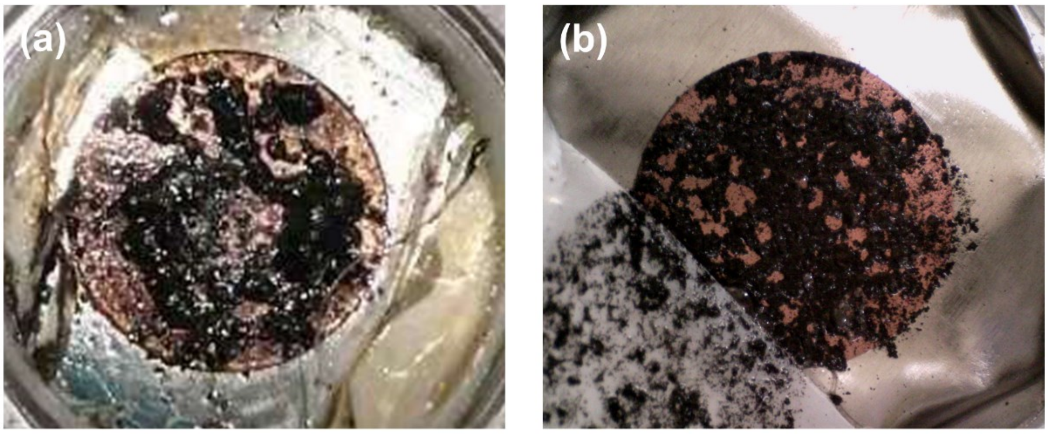

Figure S5 Images of as-prepared pure GeH and $GeCH_3$ electrodes after 100 cycles at 500 mAh g^{-1}.
